# Macrophage polarization and its impact on idiopathic pulmonary fibrosis

**DOI:** 10.3389/fimmu.2024.1444964

**Published:** 2024-07-26

**Authors:** Zhouling Ge, Yong Chen, Leikai Ma, Fangjun Hu, Lubin Xie

**Affiliations:** ^1^ Department of Respiratory Medicine, The Third Affiliated Hospital of Shanghai University (Wenzhou People’s Hospital), Wenzhou, China; ^2^ Department of Anesthesiology, The First Affiliated Hospital of Wenzhou Medical University, Wenzhou, China; ^3^ Department of Anesthesiology, Shanghai Pulmonary Hospital, Tongji University, Shanghai, China; ^4^ Department of Obstetrics and Gynecology, The Second Affiliated Hospital and Yuying Children’s Hospital of Wenzhou Medical University, Wenzhou, China

**Keywords:** idiopathic pulmonary fibrosis, macrophage polarization, M1 macrophages, M2 macrophages, fibrosis, inflammation

## Abstract

Idiopathic pulmonary fibrosis (IPF) is a lung disease that worsens over time, causing fibrosis in the lungs and ultimately resulting in respiratory failure and a high risk of death. Macrophages play a crucial role in the immune system, showing flexibility by transforming into either pro-inflammatory (M1) or anti-inflammatory (M2) macrophages when exposed to different stimuli, ultimately impacting the development of IPF. Recent research has indicated that the polarization of macrophages is crucial in the onset and progression of IPF. M1 macrophages secrete inflammatory cytokines and agents causing early lung damage and fibrosis, while M2 macrophages support tissue healing and fibrosis by releasing anti-inflammatory cytokines. Developing novel treatments for IPF relies on a thorough comprehension of the processes involved in macrophage polarization in IPF. The review outlines the regulation of macrophage polarization and its impact on the development of IPF, with the goal of investigating the possible therapeutic benefits of macrophage polarization in the advancement of IPF.

## 1 Introduction

Idiopathic pulmonary fibrosis (IPF) is the most common form of idiopathic interstitial pneumonia, characterized by chronic progressive pulmonary fibrosis associated with a steady worsening lung function, ultimately leading to respiratory failure and death (1). IPF is an age-related progressive lung disease, and its incidence increases with age. The incidence and prevalence of IPF are estimated to be in the range of 0.09-1.30 and 0.33-4.51 per 10,000 persons, respectively (2). With the global aging population, the incidence of IPF is gradually increasing yearly, and more than 5 million patients are affected worldwide (3). The high incidence and poor prognosis of IPF have gained considerable attention worldwide. Despite recent advances, several treatments including antifibrotic therapy, non-pharmacological interventions, and supportive care are available to patients with IPF, IPF is still considered to be incurable, with a median survival of only 3-5 years (4). Currently, the only effective treatment option for IPF is still lung transplantation. However, due to age and comorbidities, lung transplantation is limited to a minority of patients (5). Thus, to develop more effective therapeutics, a better understanding of the pathogenesis and pathophysiology of IPF must be gained.

IPF is characterized by tissue remodeling, fibroblast proliferation, and extracellular matrix (ECM) accumulation which involves the interstitial, distal airway, and alveolar parenchyma (6). The histopathological features of IPF are usual interstitial pneumonia, which consists of honeycombing, patchy fibrosis, fibroblastic foci, and hyperplasia of type II pneumocytes. The histopathological features of IPF consist of honeycombing, patchy fibrosis, fibroblastic foci, and hyperplasia epithelial cells (7, 8). Based on the growing number of research on IPF over the last decade, IPF is thought to be associated with both individual genetic and epigenetic factors, and IPF can be recognized as a dysregulated wound healing response in the lung. This wound-healing response is widespread and lasts for a long period in IPF, which leads to abnormal ECM accumulation and pathological lung remodeling. The repeated micro-injury of the alveolar epithelium has been proposed to be a key step in the development of fibrosis (9, 10). Although the etiology and mechanisms of IPF have been poorly elucidated, the inflammation and immune system are aberrantly activated in IPF (11).

Macrophages are considered an integral component of innate immunity that plays an essential role in immune function, tissue remodeling, and inflammatory response. Macrophages are distributed in tissues throughout the whole body and can be divided into tissue and circulating macrophages (12, 13). Macrophages are the most abundant innate immune cells in the lung tissue (~70% of immune cells), consisting of two types of tissue-resident macrophages that are characterized by their location: alveolar macrophages(AMs), which reside in the alveolar cavity, and interstitial macrophages(IMs), which exist in the interstitial areas, and they play an important role in mediating lung innate immunity and maintaining tissue homeostasis (14–17). A growing body of evidence supports the role of macrophages in regulating the pathogenesis underlying IPF (18, 19). Previous studies have shown that macrophages release various cytokines, including interleukin-1 (IL-1), interleukin-6 (IL-6), tumor necrosis factor-alpha (TNF- α), transforming growth factor-beta (TGF- β), matrix metalloproteinases (MMPs), and insulin-like growth factor 1 (IGF-1)], to regulate epithelial cell proliferation, fibroblast activation, angiogenesis, and ECM deposition, leading to pulmonary fibrosis (20–22). It has been established that macrophages are highly plastic in IPF and can differentiate into different phenotypes to modulate their functions according to the microenvironmental stimuli and signals (23, 24). Although increasing evidence has suggested the importance of macrophage polarization in IPF, the exact mechanisms for balancing the M1/M2 phenotype are still incompletely understood. Thus, further studies of the function of macrophage polarization in IPF are still required to be investigated. In this review, we summarize and critically discuss the current knowledge about macrophage polarization and the mechanisms involved in IPF.

## 2 Materials and methodology

A comprehensive literature search was conducted using the PubMed database to gather relevant studies and reviews for this review. The search terms included “macrophages”, “polarization”, “metabolic reprogramming”, “mitochondrial function”, “endoplasmic reticulum (ER) stress”, “mechanotransduction”, “epigenetic regulation”, and “signaling pathways”. These terms were combined with idiopathic pulmonary fibrosis. Both original research articles, including prospective and retrospective studies, as well as review papers, were included in the search. Cross-referencing of selected articles was performed to ensure the inclusion of all pertinent literature.

## 3 Results and discussion

### 3.1 Role of macrophages in IPF pathogenesis

Macrophages are abundant in the lung microenvironment and play a critical role in both inflammatory response and tissue repair as the first line of defense against external microbes and pathogens. In lung tissue, macrophages mainly comprise two subpopulations: alveolar macrophages (AMs) and interstitial macrophages (IMs) AMs are derived from embryonic erythromyeloid progenitor cells and fetal liver monocytes, which populate the alveolar cavity in the lung after birth, where they maintain homeostasis by self-renewal (25–28). AMs are the primary phagocytic cells and are essential in maintaining lung immunological homeostasis, expressing surface human leukocyte antigen - DR isotype (HLA-DR), leukosialin (CD43), C5a receptor 1 (CD88), sialoadhesin (CD169), macrophage mannose receptor 1 (CD206), membrane metalloendopeptidase (CD10), integrin alpha X (CD11c), fatty acid translocase (CD36), thrombomodulin (CD141), Fc-gamma receptor I (CD64), macrophage receptor with collagenous structure (MARCO), and low amounts of cluster of differentiation 14 (CD14) (29). In IPF, AMs exhibit a pro-fibrotic phenotype, producing growth factors and cytokines such as transforming growth factor-beta (TGF-β), platelet-derived growth factor (PDGF), and interleukin-13 (IL-13), which promote fibroblast proliferation and ECM deposition (30, 31). Recently, the aberrant function of AMs has been implicated in the pathogenesis of IPF. Hyperactive AMs are accumulating in the lung due to SHP-1 deficiency (32). The imbalance of the immune and metabolic status of AMs results in spontaneous inflammatory injury and pulmonary fibrosis (33–35). IMs (CXC3R1^+^, CD11b^+^, SiglecF^-^) originate from yolk sac macrophages and later to be replaced by blood monocytes (36). They are located in the interstitium between the lung epithelium and capillaries (28). IMs also participate in the fibrotic response. They contribute to the recruitment and activation of fibroblasts and myofibroblasts, cells responsible for excessive ECM production and tissue stiffening seen in IPF (37). IMs are less studied, and their predominant function is immune surveillance in the lung (38, 39). During fibrosis, the accumulation of IMs increases the permeability of blood vessels, which results in the influx of inflammatory cells into the lung (40).

The role of macrophages in IPF is complex and diverse, and macrophages in different polarization states play different functions at different stages of disease progression. During the development of IPF, the proportion and function of M1 and M2 macrophages undergo dynamic changes. In the early stage, the pro-inflammatory effect of M1 macrophages may dominate the lesion, while in the chronic stage, the anti-inflammatory and fibrotic effects of M2 macrophages are more significant. An imbalance between M1 and M2 macrophages may contribute to the progression of IPF. For example, sustained activation of M1 macrophages may lead to chronic inflammation and tissue damage, while overactivation of M2 macrophages may exacerbate fibrotic lesions (31, 41). Therefore, maintaining the balance of M1/M2 macrophages is important for disease regulation.

### 3.2 The phenotypes of macrophage polarization

Macrophages are highly plastic and heterogeneous cells. Macrophage polarization means that activated macrophages produce distinct functional phenotypes in response to stimuli and signals received from the microenvironment, which is essential for tissue repair and maintenance of homeostasis. Macrophages are commonly classified into two phenotypes: classically activated or inflammatory (M1) macrophages and alternatively activated or anti-inflammatory (M2) macrophages (42). Different phenotypes of macrophages have their own characteristics, and they are different in the expression of surface molecules and the secretion of cytokines and chemokines.

M1 macrophages are mainly induced by T helper type 1 (Th1) cytokines such as interferon γ (IFN-γ), Toll-like receptor (TLR) stimulation with agonists like lipopolysaccharide (LPS), and granulocyte-macrophage colony-stimulating factor (GM-CSF) (43, 44). M1 macrophages exert pro-inflammatory effects and anti-tumor effects by secreting numerous inflammatory mediators and cytokines such as IL-1, IL-6, interleukin-12 (IL-12), inducible nitric oxide synthase (iNOS), tumor necrosis factor-α (TNF-α), reactive oxygen species (ROS), monocyte chemotactic protein 1 (MCP-1), macrophage inflammatory protein 2 (MIP-2), and cyclooxygenase 2 (COX-2) (45–47). In addition, M1 macrophages have a strong ability to antigen presentation, which can promote the immune response of Th1 and kill pathogens of intracellular infection (48). M1 macrophages are mainly regulated by transcription factors such as nuclear factor kappa B (NF-κB), signal transducer and activator of transcription 1 (STAT1), signal transducer and activator of transcription 3 (STAT3), interferon regulatory factor 5 (IRF5), and hypoxia-inducible factor 1-alpha (HIF-1α). Excessive M1 macrophage-mediated responses may lead to tissue damage.

**Figure 1:**
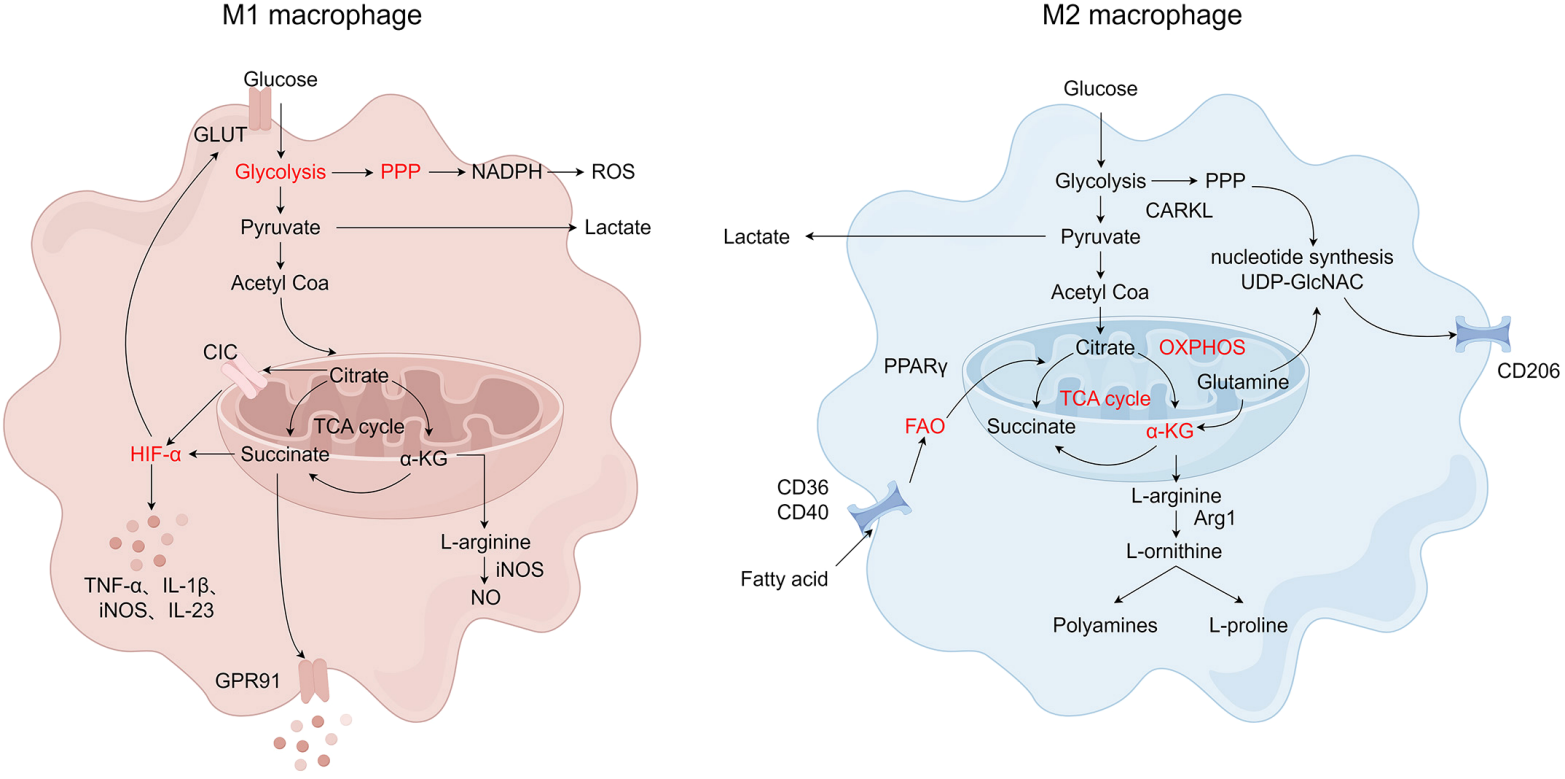
The phenotypes of macrophage polarization. Monocytes polarize into M1 and M2 macrophages. M1 macrophages are induced by IFN-γ, LPS, and TGF-α and are characterized by their pro-inflammatory functions. M2 macrophages, subdivided into M2a, M2b, M2c, and M2d, are induced by IL-4, IL-10, TGF-β, and IL-13 and are associated with anti-inflammatory responses and tissue repair. Metabolic reprogramming, mitochondrial function, endoplasmic reticulum (ER) stress, mechanotransduction, and epigenetic regulation influence the regulatory mechanisms of macrophage polarization. Created by Figdraw.

M2 macrophages are induced in response to T helper type 2 (Th2) cytokines such as interleukin-4 (IL-4), interleukin-13 (IL-13), and macrophage colony-stimulating factor (M-CSF) as well as anti-inflammatory cytokines such as interleukin-10 (IL-10) and TGF-β (47, 48). M2 macrophages are characterized by high expression of CD10, CD163, CD206, C-type lectin domain family 4 member L (CD209), and arginase 1 (Arg1) and release large amounts of the anti-inflammatory cytokines such as IL-10, TGF-β, chemokine (C-C motif) ligand 1 (CCL1), chemokine (C-C motif) ligand 13 (CCL13), chemokine (C-C motif) ligand 14 (CCL14), chemokine (C-C motif) ligand 17 (CCL17), chemokine (C-C motif) ligand 18 (CCL18), chemokine (C-C motif) ligand 22 (CCL22), and chemokine (C-C motif) ligand 24 (CCL24) to exert effects in inflammation resolution, parasite clearance, immunomodulation, angiogenesis, tumor development, and tissue repair (49–51). M2 macrophages are mainly regulated by transcription factors, such as signal transducer and activator of transcription 3 (STAT3), signal transducer and activator of transcription 6 (STAT6), interferon regulatory factor 4 (IRF4), jumonji domain containing 3 (JMJD3), peroxisome proliferator-activated receptor delta (PPAR-δ), and peroxisome proliferator-activated receptor gamma (PPAR-γ) (42). M2 macrophages can be further divided into four subtypes: M2a, M2b, M2c, and M2d, with distinct properties such as surface markers, cytokines, and functions (52). M2a macrophages are wound-healing macrophages, induced by Th2 cytokines IL-4 and IL-13 (53). They are involved in anti-inflammation, wound healing, Th2 immune response, anaphylaxis, and fibrosis, with high expression of IL-10, TGF-β, CCL17, CCL18, and CCL22 (54, 55). M2b macrophages also known as regulatory macrophages are activated by immune complexes (ICs), TLR ligands, and IL-1β, and secrete both pro- and anti-inflammatory factors, such as TNF-α, IL-1β, IL-6, and IL-10, thereby modulating the breadth and depth of immune and inflammatory responses (52, 56). M2c macrophages, also named acquired deactivation macrophages, are induced by glucocorticoids, IL-10, and TGF-β. Activated M2c macrophages secrete Arg-1, IL-10, TGF-β, CCL16, and CCL18 and play crucial roles in phagocytosis, immunosuppression, and tissue remodeling (56, 57). M2d macrophages are generally called tumor-associated macrophages (TAM), induced by TLRs and adenosine A2a receptor agonists or stimulated by IL-6 and M-CSF. These cells are characterized by the high expression of IL10, TGF-β, and vascular endothelial growth factor (VEGF) as well as the low levels of IL-12 and TGF-α and are associated with angiogenesis and tumor progression (58–60). The phenotypes of macrophage polarization are shown in **Figure 1**.

### 3.3 The regulation of macrophage polarization

#### 3.3.1 Metabolic reprogramming and macrophage polarization

Metabolic reprogramming is defined as the alterations in bioenergetic pathways in activated immune cells, including glucose metabolism, amino acid metabolism, and fatty acid metabolism (61). Recent studies have emphasized the tight link existing between metabolic reprogramming and the phenotype of macrophages (62–64). Metabolic reprogramming is an adaptation of macrophage immune regulation, focusing on the most immediate effects on the uptake and utilization of energy substrates. By changing metabolic pathways, macrophages can exhibit different phenotypic transitions. Metabolic reprogramming is not only a way to meet the energy requirements of macrophages in response to various stimuli, but also a necessary step to confer peculiar phenotypes and functions to macrophage subsets (65).

Glucose metabolism is related to inflammatory responses mediated by macrophage activation. Glucose catabolic pathways include glycolysis, pentose phosphate pathway (PPP), and mitochondrial oxidative phosphorylation (OXPHOS). Glycolysis is a conserved catabolic pathway to convert glucose into pyruvate, which plays a key role in the activation and immune function of macrophages (66, 67). Under anaerobic conditions, pyruvate is converted into lactate. In contrast, during aerobic respiration, pyruvate is transported into mitochondria to be instead converted into acetyl coenzyme A (acetyl-CoA) that enters the tricarboxylic acid (TCA) cycle to produce ATP via OXPHOS (68). M1 macrophages are characterized by increased glycolytic metabolism, PPP, and fatty acid synthesis (FAS) with decreased TCA and mitochondrial OXPHOS, while M2 macrophages display enhanced mitochondrial OXPHOS and fatty acid oxidation (FAO) with impaired PPP activity (69–72). HIF-1α is a key transcription factor that regulates glucose metabolism and stimulates glycolytic gene expression during hypoxic conditions (73). HIF-1α induces some glycolytic enzymes, including hexokinase, phosphofructokinase, and glucose transferase (GLUTs). On the other hand, HIF-1α is also associated with the increase of M1-related genes such as TNF-α, IL-1β, iNOS, and IL-23. As mentioned above, HIF-1α promotes M1 macrophage polarization by inducing glycolysis gene expression and GLUTs in response to inflammatory stimulation (74, 75). Pyruvate dehydrogenase kinase 1 (PDK1) and pyruvate kinase isozymes M2 (PKM2) are key regulatory enzymes in glucose metabolism (76, 77). Inhibition of PDK1 and activation of PKM2 inhibit glycolysis to diminish LPS-induced pro-inflammatory M1 macrophages and promote the typical characteristics of M2 macrophages (77, 78). Furthermore, 2-deoxy-d-glucose (2-DG) reduces the production of IL-10 and other polarization-associated genes to inhibit the proinflammatory phenotype of M1 macrophages by blocking glycolysis (79, 80). 2-DG also attenuates enhanced mitochondrial respiration and reduces the expression of M2 phenotypic markers, such as Arg1, chitinase-like protein 3 (YM-1), found in inflammatory zone 1 (FIZZ-1), and CD206 (81).

The PPP, also known as the hexose monophosphate shunt or the phosphogluconate pathway, is a branched pathway from glycolysis. The PPP is the major source of NADPH, which is a redox cofactor for ROS production via nicotinamide adenine dinucleotide phosphate hydrogen (NADPH) oxidase and is required for both the generation of the antioxidant glutathione and lipid synthesis (61, 82, 83). Carbohydrate kinase-like protein (CARKL), also known as sedoheptulokinase (SHPK), is a sedoheptulose kinase of PPP that regulates the formation of sedoheptulose-7-phosphate (S7P) (84). CARKL is down-regulated in response to LPS in M1 macrophages and highly expressed by IL-4 stimulation in M2 macrophages (85). The overexpression of CARKL in macrophages leads to M1 polarization defect and inhibits inflammatory response (86). Meanwhile, the overexpression of CARKL in M2 polarization leads to ribose-5p production by enhancing the non-oxidative steps of PPP, which is necessary for nucleotide and uridine diphosphate N -acetylglucosamine (UDP-GlcNAC) synthesis (85). UDP-GlcNAC is necessary for N-glycosylation, which is required to modify different cell surface proteins (such as CD206) that are heavily expressed in M2 macrophages (87). Therefore, the regulation of glucose metabolism in macrophages determines M1/M2 polarization and regulates their immune function.

Fatty acid metabolism, including FAO and FAS, is composed of anabolic and catabolic processes that generate diverse metabolic intermediates to facilitate energy storage, the biosynthesis of membrane lipids, and the production of signaling molecules (88). The metabolic synthesis of fatty acids, particularly the synthesis of fatty acids, is closely related to the pro-inflammatory function of macrophages (89, 90). In the process of *de novo* synthesis of fatty acids, carbon atoms from glucose or amino acids are converted to fatty acids, which tightly couple glucose and lipid metabolism (91). *De novo* FAS is suggested to fuel FAO in M2 macrophages (92). While LPS/IFN-γ promotes M1 macrophages to synthesize fatty acids, M2 macrophages are known to increase fatty acid uptake and catabolism through catabolic FAO or β-oxidation (93). The main fatty acid source for M2 macrophages is the uptake of triacylglycerol substrates through CD36 and their subsequent lipolysis via lysosomal acid lipases (92). PPAR‐γ, as a number of the nuclear receptor family of ligand-inducible transcription factors, is an important transcription factor regulating macrophage polarization (94). The PPARγ family of transcriptional coactivators including PPARγ coactivator-1α (PGC-1α) and PPARg-coactivator-1β (PGC-1β) also induce FAO and mitochondrial biogenesis transcriptionally to participate in M2 polarization of macrophages (95, 96). Liu et al. demonstrated that S100A4 enhances M2 macrophage polarization by controlling PPAR-γ-dependent FAO induction via up-regulating CD36/CPT1 (97). Liu et al. showed that the CD40 signal can drive M2 macrophage polarization by exploiting FAO (98). However, after treatment with an anti-CD40 monoclonal antibody, FAO could promote M1 polarization of macrophages instead of supporting M2 polarization (98).

**Figure 2:**
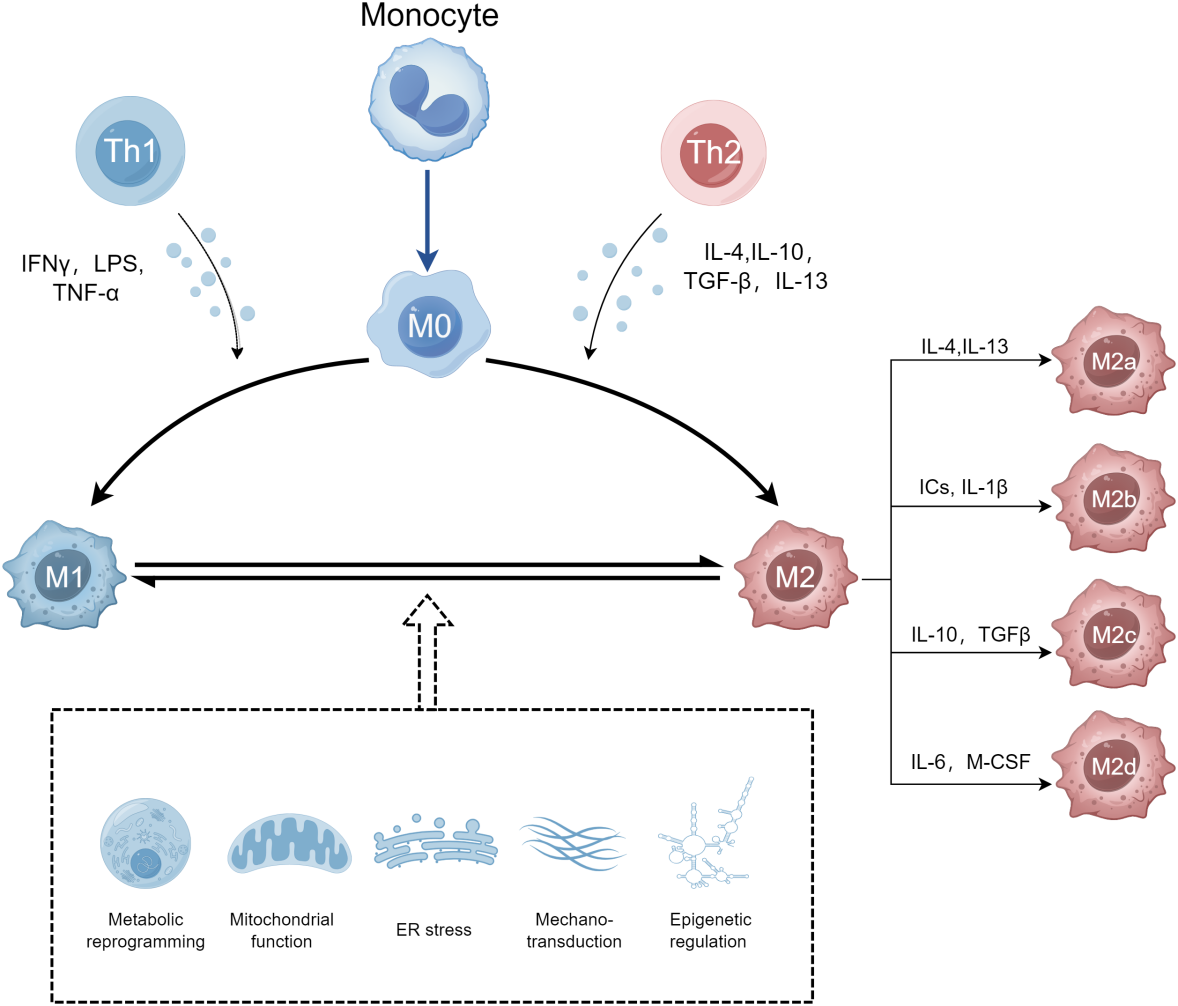
The metabolic reprogramming of macrophages. M1 and M2 macrophages have different metabolic pathways. M1 macrophages rely on glycolysis and the pentose phosphate pathway (PPP), producing reactive oxygen species (ROS) and inflammatory mediators. M2 macrophages primarily use oxidative phosphorylation (OXPHOS) and fatty acid oxidation (FAO) for energy production, supporting anti-inflammatory functions and tissue remodeling. Created by Figdraw.

Amino acid metabolism also has an important influence on macrophage polarization. M1 and M2 macrophages show different arginine metabolism characteristics. M1 macrophages metabolize arginine to nitric oxide (NO) and citrulline with high levels of iNOS, while M2 macrophages metabolize arginine to ornithine and polyamines via enzyme arginase, ornithine decarboxylase, and spermidine oxidase (99). In addition, creatine, a metabolite of arginine, also plays a part in macrophage polarization. The uptake of creatine reprogrammed macrophage polarization by inhibiting IFN-γ-JAK-STAT1 signaling and suppressing the expression of TNF-α, while promoting IL-4-STAT6-activated arginase expression (100, 101). The difference between these two arginine metabolic pathways forms the basis of M1/M2 polarization of immune response. During macrophage activation, glutamine metabolism modulates the polarization of macrophages. Glutamine is converted to glutamate by glutaminase and then transformed into α-ketoglutarate (α-KG) via glutamate dehydrogenase, which enters the TCA cycle as a key intermediate metabolite to provide energy (102). α-KG is essential for increasing FAO as well as OXPHOS during M2 macrophage activation. α-KG promotes M2 macrophage polarization through the SUMO‐specific protease 1 (SENP1) - Sirtuin 3 (Sirt3) axis in bone marrow-derived macrophages (BMDMs) (103). In macrophages, α-KG induces the M2 phenotype through JMJD3-dependent demethylation of histone 3 lysine 27 (H3K27). An increased α-KG: succinate ratio further promotes M2 macrophage polarization, while a decreased α-KG: succinate ratio induces the M1 macrophage phenotype (104). Also, α-KG inhibits the activation of the NF-κB pathway and destabilizes HIF-1α as the substrate for prolyl hydroxylases, thereby restricting the M1 macrophages activation (104, 105). Glutamine also promotes M2 macrophage polarization through the glutamine–UDP-GlcNAc pathway. It has been found that more than half of the nitrogen in UDP-GlcNAc is derived from glutamine. As a sugar donor affecting the activation of M2 macrophages, UDP-GlcNAc plays an important role in the polarization of M2 macrophages because it is responsible for the glycosylation of M2 marker proteins (87, 106). Several other amino acids, including serine, glycine, tryptophan, and branched-chain amino acids, also regulate the activation states of macrophages (107). Serine and glycine participate in redox homeostasis during M1 polarization, and the serine synthesis pathway is related to the response of macrophages to LPS/IFN-γ and IL-4 (108–111). The level of citrulline in macrophages drops rapidly after IFN-γ and/or LPS stimulation, which is necessary for efficient proinflammatory signaling activation. And argininosuccinate synthetase (ASS1) regulates inflammatory macrophage activation and antimicrobial defense by reducing citrulline (112). The metabolic reprogramming of macrophages is shown in **Figure 2**.

#### 3.3.2 Mitochondrial function and macrophage polarization

Mitochondria is a crucial double-membrane organelle in all eukaryotic cells and serves as a central hub in cellular metabolism, innate and adaptive immune responses, homeostasis, and stress responses, regulating cell growth, division, differentiation, and death (113, 114). Multiple signaling pathways intersect and collaborate to govern the interconnected processes of mitochondrial energetics, biogenesis, ROS production, preservation and repair of mitochondrial DNA (mtDNA), and mitophagy (115). Studies have shown that mitochondrial function is critical in shaping macrophage polarization (45, 116).

The metabolic and physiological changes of mitochondria may be the basis of macrophage activation. Several important metabolic pathways play roles in mitochondria, including FAO, TCA cycle, and OXPHOS via electron transport chain (ETC). In M1 macrophages, two breaks on the TCA cycle and inhibition of parts of ETC in the mitochondria were demonstrated, leading to the accumulation of itaconate, citrate, and succinate (117). Meanwhile, M2 macrophages are more dependent on OXPHOS and have a complete TCA cycle, which provides the substrates for the complexes of ETC (65). The metabolic difference between M1 and M2 macrophages leads to the difference in their ability to produce ROS. Mitochondrial ROS induces and maintains M1 polarization through mitogen-activated protein kinase (MAPK), NF-κB, STAT1, and STAT6 signaling pathways and induces transcription of pro-inflammatory genes, such as iNOS, IL-6, TNF-α, and IL-1β (118, 119). Furthermore, mitochondrial ROS plays an important role in NLRP3 (NOD, LRR, and Pyrin domain-containing protein 3) inflammasome initiation, and many studies have shown that NLRP3 inflammasome can modulate macrophages to M1 phenotype (120, 121). NADPH oxidase (NOX), the outer mitochondrial membrane-bound enzymes, and other cellular activities in mitochondria such as autophagy are also involved in regulating ROS levels. NOX1, NOX2, and NOX4 are essential for the differentiation of monocytes into macrophages and polarization of macrophages. Another source of mitochondria-related ROS is monoamine oxidase (MAO), including the two subtypes: MAO-A and MAO-B. MAO-A induced M2 macrophages polarization by promoting the JAK-STAT6 pathway (122). The formation of MAO-B-dependent ROS leads to mitochondrial dysfunction and activation of NF-κB, resulting in overexpression of NLRP3 and pro-IL-1β (123). In addition, uncoupling protein 2 (UCP2) located in the inner membrane of mitochondria participates in macrophage polarization by mtROS (124). The inhibition of autophagy promotes the production of macrophage migration inhibitory factor (MIF) associated with ROS, which is beneficial to the polarization and paracrine effect of M1 macrophages on pro-inflammatory response (125).

TCA cycle metabolites, including succinate, itaconate, α-KG, and citrate, play important roles in the modulation of macrophages. In M1 macrophages, citrate is exported to the cytoplasm through the transport activity of mitochondrial citrate carrier (CIC), which leads to the increase of HIF-1α expression (126). HIF-1α also up-regulats the immune response gene 1 (IRG1) for itaconate production (127). CIC inhibition can restrain glycolysis and enhance mitochondrial oxidation, which leads to the transformation of M1 macrophages into M2 macrophages (128). Another important role of citrate is its conversion to acetyl-CoA via adenosine triphosphate citrate lyase (ACLY) (129). ACLY is regulated by the Akt-mTORC1 axis, and IL-4 activates the Akt-mTORC1 signaling pathways, increasing histone acetylation and inducting M2 gene expression, resulting in M2 macrophage activation (130). However, a recent study shows that ACLY may not be the primary regulator of nucleocytoplasmic acetyl-CoA and IL-4-induced polarization in human macrophages (131).

Itaconate is synthesized from cis-aconitate in the TCA cycle by IRG1 in macrophages after LPS stimulation (132). Itaconate has been demonstrated to exert anti-inflammatory effects by inhibiting the production of succinate dehydrogenase (SDH), a key enzyme in the TCA cycle, and Complex II of the ETC, resulting in increased accumulation of succinate and decreased levels of mitochondrial ROS, which inhibits the release of pro-inflammatory cytokines (133, 134). Itaconate and its derivate 4-octyl itaconate (4-OI) contribute to stabilizing the levels of nuclear factor erythroid 2-related factor 2 (NRF2) by alkylating multiple cysteine residues of kelch-like ECH-associated protein 1 (KEAP1) to enhance anti-inflammatory and antioxidant response in macrophages (135, 136). Itaconate and its derivative dimethyl itaconate (DI) also exerte anti-inflammatory effects by inhibiting the IκBζ-ATF3 inflammatory axis (137). Furthermore, itaconate inhibits M2 macrophages polarization by inhibiting JAK1 and STAT6 phosphorylation (138).

The TCA cycle metabolite succinate accumulates in LPS-stimulated macrophages. Accumulated succinate is oxidized to fumarate by SDH and increases mitochondrial ROS production through reverse electron transport (RET) from complex II to complex I and PHD inhibition, resulting in stabilizing HIF-1α and promoting IL-1β production in proinflammatory macrophages (139–141). Succinate can also play a signaling role outside the cell through binding to G-protein-coupled receptor 91 (GPR91), which is also named succinate receptor 1 (SUCNR1). During inflammation, macrophages release succinate into the extracellular milieu. Succinate activates macrophage GPR91 receptor to sustain the proinflammatory phenotype and lead to IL-1β production in an autocrine manner (142). However, tumor-derived succinate in the TME induces macrophage polarization into TAM by activating GPR91 (143).

mtDNA is an important component of mitochondrial function and is crucial for the production of cellular energy. Mitochondrial dysfunction leads to the release of mtDNA into the cytosol and outside cells. Upon entering the cytosol, mtDNA can trigger responses by activating four innate immune receptors: cytoplasmic cyclic GMP-AMP synthase (cGAS), TLR9, absent in melanoma 2 (AIM2), and NLRP3. Cytosolic mtDNA can further trigger inflammatory responses by activating cGAS-stimulator of interferon genes (STING)-IRF3-dependent signaling pathway (144). mtDNA contains unmethylated CpG motifs, which are recognized by TLR9, and mtDNA-bound TLR9 in macrophages initiates MyD88-dependent immune responses which activates MAPK and NF‐κB signaling pathway to induce the release of pro-inflammatory factors (145–148). In macrophages, mtDNA also contributes to NLRP3 and AIM2 inflammasome activation, which leads to the activation of caspase-1 and subsequently the maturation of pro-IL-1β and pro-IL-18 (149, 150).

Mitophagy refers to the process of selective elimination of damaged and dysfunctional mitochondria from macrophages via autophagy for mitochondrial quality control and homeostasis. Mitophagy is regulated by phosphatase and tensin homolog (PTEN) -induced putative kinase 1 (PINK1) and the ubiquitin ligase parkin. The lack of PINK1/parkin results in macrophage reprogramming to M2 phenotype and promotes kidney fibrosis (151), and the overexpression of PINK1 reverses the polarization of macrophages to the M1 phenotype (152). Moreover, Esteban-Martinez et al. suggested that BNIP3L/NIX-dependent mitophagy regulates metabolic reprogramming toward glycolysis which promotes macrophage polarization toward the M1 phenotype (153). Mitophagy can be inhibited with 3-methyladenine (3-MA) resulting in macrophage polarization towards the M1 phenotype, while the induction of mitophagy with rapamycin can enhance the M2 phenotype in macrophages by inhibiting the production of mtROS and NLRP3 inflammasome activation (154, 155).

Mitochondrial structure and dynamics also play key roles in macrophage polarization. Mitochondrial dynamics, consisting of fusion and fission, and mitophagy jointly maintain mitochondrial homeostasis and functions. In mammals, mitochondrial fusion machinery is controlled by two Dynamin family guanosine triphosphatases (GTPases): mitofusin 1 (MFN1) and mitofusin 2 (MFN2) that mediate the fusion of outer membrane and optic atrophy protein 1 (OPA1) for the inner membrane (156, 157). Fission is controlled by dynamin-related protein 1 (DRP1) and its four outer membrane proteins: fission 1 (FIS1), mitochondrial fission factor (MFF), and mitochondrial division (Mid) 49 and 51 (158, 159). In IL-4-stimulated macrophages, mitochondrial fusion stimulates interactions between ETC complexes in favor of OXPHOS and FAO, while in M1 macrophages fission leads to crest expansion, which inhibits ETC efficiency and enhances aerobic glycolysis (160). Li et al. found that XBF promoted mitochondrial fusion by upregulating MFN1 to inhibit NF-κB pathways, and subsequently inhibited pro-inflammatory macrophage polarization (161). Notably, MFN2 is found to be critical for LPS-induced ROS production and pro-inflammatory signaling, as well as other pro-inflammatory roles of macrophages (162). Divya et al. found that the downregulation of MFN2 but not MFN1 leads to the polarization of macrophages toward the M2 phenotype to promote renal fibrosis through mechanisms that inhibit macrophage mitophagy and dysfunctional mitochondrial dynamics (163). OPA1 is a mitochondria-shaping protein involved in mitochondrial fusion, cristae biogenesis, and respiration. OPA1 deficiency in macrophages leads to the increase of αKG/succinate ratio and defective activation of NF-κB signaling that impairs the M1 macrophage phenotype (164). DRP1 has been reported to play a central role in the pro-inflammatory macrophage response. Sustained activity of DRP1 causes macrophages to exhibit proinflammatory activity even in the absence of LPS, whereas loss or inhibition of DRP1 attenuates M1 polarization. In BMDMs, DRP1 knockdown initiated NLRP3 inflammasomal activation and IL-1β secretion, while inducing mitochondrial fission attenuated NLRP3 inflammasomal assembly and activation (165). DRP1-mediated mitochondrial fission can also promote the secretion of TNF-α and the production of ROS (166, 167).

#### 3.3.3 Endoplasmic reticulum stress and macrophage polarization

Endoplasmic reticulum (ER) stress refers to an imbalance of the ER homeostasis resulting in the accumulation of misfolded proteins and the activation of the unfolded protein response (UPR). The UPR is mediated by three ER-transmembrane sensors: inositol-requiring enzyme 1α (IRE1α)-X box binding protein-1(XBP1), protein kinase-like endoplasmic reticulum kinase (PERK)-eukaryotic translation initiation factor 2α(eIF2α)- activating transcription factor 4(ATF4), and activating transcription factor 6 (ATF6), which are regulated by the ER chaperone BiP/GRP78 (168). Studies have shown that ER stress is involved in the process of macrophage polarization (169, 170). The knockdown of IRE1α in macrophages inhibits the production of IL-6, TNF-α, and IFN-β. In addition, the IRE1α-XBP1 pathway regulates the production of IL-1β and TNF-α by GSK-3β activation (171, 172). The inhibition of the IRE-1α/XBP-1 signaling pathway suppresses M1 polarization and alleviates LPS-induced lung injury (173). PERK-eIF2α-ATF4 pathway can also participate in and regulate macrophage polarization. The knockdown of PERK promotes the polarization into M2 macrophages via the STAT1 and STAT6 pathways (174).

It is known that ATF4 is involved in SFA-induced NF-κB activation and binds the IL-6 promoter directly to exert its proinflammatory effects (175, 176). On the other hand, the ATF6 synergizes with TLR stimulation and enhances NF-κB signaling in macrophages (177). Wang et al. found that microcystin-LR is selectively absorbed by macrophages in lung tissue and binds to GRP78, attenuating M2 polarization of macrophages by reducing p-PERK, p-IRE1α, and cleaved ATF6 (178). ER stress is involved in IL-10-mediated macrophage polarization. Reduced IL-10 secretion activates ER stress, thus inhibiting M2 polarization (179). Additionally, ER stress is reported to be another source of ROS (180). ER stress can induce the secretion of septin 2 (SEPT2), and SEPT2, as a negative regulator, inhibits the polarization of M1 macrophages. The imbalance of negative feedback regulation leads to the accumulation of UPR, thus accelerating the polarization of M1 macrophages and the release of inflammatory factors (181).

Recent studies have highlighted the involvement of tripartite motif-containing 29 (TRIM29) and PERK in this process. TRIM29 is uniquely and highly expressed in mouse alveolar macrophages (AMs) and degrades NEMO, thereby inhibiting the NF-κB signaling pathway and reducing the production of pro-inflammatory cytokines, which plays a crucial role in maintaining immune homeostasis in the lungs (182). PERK, one of the key sensors of ER stress, has been reported to control M2 polarization by regulating metabolic reprogramming (183). Inhibition of PERK suppresses M2 macrophage activity and enhances anti-tumor immunity, suggesting that targeting PERK could be a potential therapeutic strategy for improving cancer treatment and modulating chronic inflammation. Recent findings have suggested that TRIM29 can exacerbate viral myocarditis by promoting PERK-mediated ER stress, apoptosis, and inflammation, while TRIM29 deficiency protects against these effects (184). Given the critical role of macrophage polarization in the development of IPF, it is plausible that TRIM29 could utilize PERK-mediated ER stress immune signaling to regulate AM M2 polarization. This regulation might, in turn, control the pathogenesis of IPF. Targeting the TRIM29-PERK axis could provide new therapeutic strategies for managing IPF by modulating the ER stress response and macrophage polarization.

#### 3.3.4 Mechanotransduction and macrophage polarization

Mechanotransduction is the process by which living organisms convert external physical forces into biochemical signals, enabling cells to sense and respond to their mechanical environment (185). Recent studies have highlighted the pivotal role of mechanotransduction in regulating macrophage polarization, thereby influencing immune responses and tissue repair (186). The physical properties of the cellular microenvironment, such as matrix stiffness, topology, and interstitial flow, significantly impact macrophage behavior and phenotype. The stiffness of the ECM can influence macrophage polarization through the activation of the Yes-associated protein (YAP) and transcriptional coactivator with PDZ-binding motif (TAZ) signaling pathways (187, 188). Studies have demonstrated that macrophages promote an M1 phenotype when cultured on stiffer substrates, characterized by higher expression levels of pro-inflammatory cytokines and surface markers (189, 190). Conversely, softer matrices are more conducive to promoting an M2 phenotype, associated with tissue repair and resolution of inflammation (190). In contrast, other studies observed that stiffer matrices promoted an M2 phenotype with increased expression of anti-inflammatory markers (191, 192). These discrepancies may arise from different experimental conditions, highlighting the sensitivity of macrophages to material stiffness and the need for further studies to fully understand the role of matrix stiffness on macrophage phenotype commitment. Topography such as surface roughness can influence macrophage polarization through an integrin-mediated signaling pathway: the surface roughness can induce src-mediated focal adhesion kinase (FAK) phosphorylation, leading to nuclear translocation of extracellular signal-regulated kinases 1 and 2 (ERK1/2) and increased secretion of proinflammatory cytokines in macrophages (193–195). Additionally, substrate stiffness and topography might activate the NF-κB signaling pathway in macrophages (196, 197). Interstitial flow, the movement of fluid through the ECM of tissues, can influence macrophage polarization in several ways. Interstitial flow can exert mechanical forces on cells, including shear stress and pressure, which can alter cell signaling pathways and influence macrophage polarization. Li. et al. found that interstitial fluid flow can enhance macrophage polarization towards an M2 phenotype through integrin/Src-mediated mechanotransduction pathways involving increased expression of phosphorylated STAT3/6 and CD206 (198).

Macrophage polarization is also influenced by spatial confinement and cell shape. Spatial confinement, as a component of the cellular microenvironment, can significantly impact macrophage polarization by modulating cellular processes such as cytokine production, phagocytosis, and gene expression. Spatial confinement in densely packed tissue environments may downregulate the inflammatory response of macrophages, likely through alterations in gene expression related to actin polymerization and the subsequent nuclear translocation of transcription factors such as myocardin-related transcription factor A (MRTF-A) (199). Spatial confinement of macrophages can also “tame down” their phagocytic potential and suppress late LPS-activated transcriptional programs via mechanomodulating chromatin compaction and epigenetic alterations (HDAC3 levels and histone 3 lysine 36 dimethylation), which influences the expression of genes involved in proinflammatory cytokine secretion (e.g., IL-6, CXCL9, and iNOS) (186, 199).

Alterations in cell shape are closely associated with changes in macrophage function. In macrophages, cell shape changes are induced by both physical cues, such as topography, stretch, and substrate stiffness, and by soluble factors like cytokines. Studies have shown that cyclic biaxial stretch increases the spread of cell area, while uniaxial stretch leads to cellular elongation (200, 201). Substrate stiffness has been observed to affect macrophage morphology, with macrophages appearing more spread and flattened on rigid substrates and more rounded on softer ones (202). Soluble factors like LPS and IFN-γ tend to induce a more circular and flattened morphology in murine BMDMs, whereas M2-inducing cytokines like IL-4 and IL-13 lead to elongated cell shapes (203, 204). This change in morphology is associated with macrophage polarization. For instance, using micropatterning techniques to control cell shape has demonstrated that elongated cell morphology can promote macrophage polarization towards an M2 phenotype, characterized by increased expression of M2 markers like arginase-I, CD206, and YM-1. Furthermore, cell shape modulation can influence the macrophage response to soluble factors, either enhancing the expression of M2 markers or dampening M1 activation (205).

Macrophages can also express mechanosensitive ion channels to modulate cellular activity through the gating of soluble ions. The Piezo1 ion channel is a critical component of cellular mechanosensation and has been implicated in various cellular processes. Mechanical forces activate Piezo1 channels, allowing calcium ions (Ca^2+^) to enter the cell and trigger downstream pathways (206). In macrophages, activation of the Piezo1 pathway can significantly influence their polarization states, potentially modulating their roles in inflammation, tissue repair, and disease progression. For instance, mechanical cues transmitted through the Piezo1 channel can promote macrophages to an M2 phenotype, which is associated with tissue healing and resolution of inflammation (207). Conversely, under certain conditions, Piezo1 activation might also contribute to M1 macrophages polarization, depending on the cellular microenvironment and the interplay with other signaling pathways (208). Another category of ion channels found on macrophages is transient receptor potential (TRP) family channels, including TRPV2, TRPV4, TRPC6, and TRPM7, which are known to be sensitive to mechanical stimuli such as alterations in membrane stretch, pressure, and shear stress (209). Activation of TRPV4 by mechanical forces can lead to calcium influx into the cell, which is a pivotal step in the signaling cascade that governs macrophage polarization (210). Matrix stiffness promotes M1 Macrophage polarization in a TRPV4-dependent Manner. The reintroduction of TRPV4 into TRPV4 knockout (KO) macrophages significantly upregulated the expression of M1 markers (211).

#### 3.3.5 Epigenetic regulation and macrophage polarization

Epigenetics refers to the study of heritable changes in gene expression that do not involve alterations in the DNA sequence itself. These changes are mediated by various mechanisms, including DNA methylation, histone modifications, and non-coding RNAs, which can influence cellular functions and contribute to development, disease, and inheritance (212). Epigenetics is essential in regulating macrophage polarization towards pro-inflammatory M1 or anti-inflammatory M2 phenotypes, affecting immune responses and tissue repair (213).

DNA methylation, the addition of a methyl group to the 5’ carbon of the cytosine ring within CpG dinucleotides, serves as a key epigenetic marker that typically represses gene expression (214). This modification is catalyzed by DNA methyltransferases (DNMTs), including DNMT1, DNMT3A, and DNMT3B, while ten-eleven translocation (TET) proteins remove the modification (215, 216). Research indicates that the dynamic balance between DNA methylation and demethylation plays a pivotal role in macrophage polarization. DNMTs add methyl groups to CpG dinucleotides, suppressing gene expression associated with the opposing macrophage phenotype. Conversely, TET enzymes demethylate these regions, enabling the expression of genes required for the alternative phenotype. This balance ensures that macrophages can respond adaptively to environmental cues, adjusting their polarization state as needed. For instance, DNMT1 has been shown to mediate promoter hypermethylation of the SOCS1 gene, activating the JAK/STAT pathway and promoting M1 polarization (217). DNMT3b has been highlighted for its role in targeting and inhibiting the promoter of PPARγ, a critical regulator of M2 macrophage polarization, thus promoting an M1 phenotype (218, 219). DNMT3a and DNMT3b-mediated the hypermethylation of PSTPIP2 caused a mixed induction of hepatic M1 and M2 biomarkers in CCL4-induced hepatic fibrosis (HF) mice (220). Furthermore, studies have demonstrated that inhibiting DNMT1-mediated PPARγ1 promoter DNA methylation with 5-aza-2′-deoxycytidine in obesity models significantly promotes macrophage M2 phenotype (221). TET2 increases IL-1β, IL-6, and Arg1 mRNA expression in BMDMs and recruits histone deacetylase 2 (HDAC2) to specifically repress transcription of IL-6 via histone deacetylation (222, 223). In addition, TET2 deficiency in murine macrophages increases JNK1 activation-mediated NLRP3 inflammasome activation and BRCC3-mediated NLRP3 deubiquitylation (224).

Histone modifications are crucial epigenetic mechanisms that regulate gene expression by altering chromatin structure and accessibility. These modifications include methylation, acetylation, phosphorylation, sumoylation, ubiquitination, lactylation, and so on (225–227). The most studied modifications in the context of macrophage polarization are histone methylation and acetylation, which are initiated by histone methyltransferases (HMTs) and demethylases (HDMs), acetyltransferases (HATs) and deacetylases (HDACs). Methylation of histones can either activate or repress gene expression depending on the number and location of the methyl groups. Histone acetylation, mediated by HATs, generally marks transcriptionally active regions, whereas HDACs remove these acetyl groups, leading to chromatin condensation and gene silencing. For instance, methylation of H3K27, often associated with gene repression, can be dynamically regulated by enzymes such as EZH2 (enhancer of zest homolog 2), which adds methyl groups, and JMJD3, which removes them, to modulate genes critical for macrophage polarization (228, 229). HAT p300 is known to acetylate histones H3 and H4, leading to the transcription of M2 phenotype genes by promoting chromatin accessibility (230, 231). Histone deacetylases, particularly HDAC1, HDAC2, and HDAC3, are involved in removing acetyl groups from histones, leading to a more compact chromatin structure and gene repression. In macrophages, HDAC activity can suppress the expression of M2 markers while promoting M1 genes expression (232–234).

Non-coding RNAs (ncRNAs) are RNA molecules that do not code for proteins but play crucial roles in regulating gene expression at the transcriptional and post-transcriptional levels. These include microRNAs (miRNAs), long non-coding RNAs (lncRNAs), and circular RNAs (circRNAs), which are involved in macrophage polarization based on their ability to drive M1 or M2 polarization (235). miRNAs are short, 18-25 nucleotide-long non-coding small RNA species that regulate gene expression by binding to the 3’ untranslated regions (UTRs) of target mRNAs, leading to the inhibition of translation. Multiple miRNAs have been identified as crucial regulators of macrophage polarization by targeting key signaling molecules and transcription factors involved in the M1/M2 polarization pathways. For instance, miR-204-3p and miR-26b-5p support M2 polarization by inhibiting TLR signaling and pro-inflammatory cytokine release, while miR-27a-3p induces the M1 polarization via the PPARγ/NF-κB/PI3K/AKT signaling pathway (236–238). LncRNAs are a class of non-coding RNAs longer than 200 nucleotides. LncRNA NEAT1 promotes Bruton’s tyrosine kinase (BTK) transcription by downregulating the transcription factor Krüppel-Like Factor 4 (Klf4), which subsequently leads to the activation of the NF-κB pathway, NLRP3 inflammation and M1 polarization in BMDMs, whereas lncRNA NEAT1 from endothelial cells enhances M2 polarization via DDX3X/NLRP3 axis (239, 240). Some lncRNAs can also act as “sponges” for miRNAs to regulate macrophage polarization. For example, lncRNA TUG1 induces M1 macrophage polarization via the miR-1192/TLR3 axis (241). CircRNAs, a novel class of ncRNAs with a closed-loop structure, have recently been implicated in the regulation of macrophage polarization. CircRNA Cdyl promotes M1 polarization by inhibiting IRF4 entry into the nucleus (242). Some circRNAs can also modulate macrophage polarization by sequestering miRNAs or interacting with RNA-binding proteins. CircRNA ATP8A1 induces M2 polarization via miR-1-3p/STAT6 axis, and circATP2B4 induces M2 polarization by sponging miR-532-3p (243, 244).

In summary, the mechanisms of DNA methylation, histone modifications, and non-coding RNAs collectively contribute to the complex regulatory network that determines macrophage polarization. This dynamic and reversible epigenetic landscape allows macrophages to respond to environmental cues with precise control over gene expression, facilitating their roles in immunity, tissue homeostasis, and diseases.

#### 3.3.6 The mechanism of macrophage polarization

Macrophage polarization is regulated by a complex interplay of signaling pathways, each contributing to the dynamic balance between pro-inflammatory and anti-inflammatory macrophage phenotypes. The NF-κB pathway emerges as a central regulator of the inflammatory response, with its activation steering macrophages towards an M1 phenotype. Inhibition of NF-κB signaling has been shown to skew macrophages towards an M2 phenotype (245). Similarly, S100A9 gene deficiency improves LPS-induced acute lung injury in mice by inhibiting the TLR4/MyD88/NF-κB signaling pathway and inhibiting M1 macrophage polarization (246). The impact of the JAK/STAT pathway on macrophage polarization has been highlighted in recent years, with JAK inhibitors reducing the secretion of proinflammatory cytokines IFN-γ and TNF-α, thereby shifting macrophage polarization towards an M2 phenotype (247). Furthermore, tumor-derived miR-6794-5p induced M2 polarization to evade immune surveillance by activating the JAK1/STAT3 pathway (248). Another critical pathway is the PI3K/AKT signaling axis, which influences macrophage polarization towards both M1 and M2 phenotypes. Song et al. highlighted the significant activation of the PI3K/AKT/HIF-1α signaling pathway in promoting macrophage polarization towards the M2 phenotype (249). Zhang et al. identified the PI3K/AKT signaling pathway as a key element in modulating M1 macrophage polarization (250). Research into lung fibrosis has highlighted the roles of Notch and TGF-β/Smad signaling pathways in macrophage polarization (251). The Notch signaling pathway promotes M1 polarization to enhance pro-inflammatory responses, while its inhibition can favor M2 polarization, indicating its versatile influence on macrophage functionality in various disease contexts (252, 253). TGF-β is known for its anti-inflammatory properties and typically promotes the polarization of macrophages towards the M2 phenotype. TGF-β/Smad signaling predominantly promotes M2 macrophage polarization (254). Upon activation by TGF-β, this pathway involves the phosphorylation of Smad2 and Smad3, their association with Smad4, and subsequent nuclear translocation of the complex to regulate gene expression that favors M2 polarization (255). In addition, both TGF-β and Smads signaling can regulate macrophage polarization individually (256, 257). Peroxisome Proliferator-activated receptors (PPARs) are a group of nuclear receptor proteins that play essential roles in regulating cellular metabolism, development, and inflammation. PPARs are now recognized as important determinants of macrophage polarization. PPAR-α activation is associated with the upregulation of genes involved in FAO, which can modulate macrophage function to an M2 phenotype, thus reducing pro-inflammatory responses and enhancing the resolution of inflammation (258). The role of PPAR-δ in Kupffer cells has been shown to influence their polarization towards an M2-like state by reducing sensitivity to IL-4 (259). PPAR-δ also has a pivotal role in regulating lipid metabolism and inflammatory responses in macrophages. Activation of PPAR-δ leads to enhanced clearance of apoptotic cells and promotes an M2 phenotype (260). PPAR-γ is well-known for its strong influence in inducing an M2 phenotype in macrophages, PPAR-γ activation leads to increased expression of genes associated with fatty acid storage and glucose metabolism, which are vital for the functioning of M2 macrophages (97, 261, 262). PPAR-γ also counteracts pro-inflammatory signals such as those mediated by NF-κB, thus promoting a switch towards an M2 phenotype (263).

### 3.4 The role of macrophage polarization in IPF

. The pathogenesis of IPF is complex, involving a variety of cellular players and signaling pathways. Among these, macrophages play a critical role due to their plasticity and ability to polarize into different functional phenotypes, primarily pro-inflammatory M1 and anti-inflammatory M2 macrophages (264).

Typically, M1 macrophages exhibit pro-inflammatory functions, producing cytokines like IL-1β and TNF-α, which are crucial in the initial stages of IPF. They help in pathogen clearance and orchestrate the inflammatory responses crucial for the subsequent fibrotic processes (19). However, persistent activation of M1 macrophages can lead to chronic inflammation and tissue damage (47). Studies have demonstrated elevated levels of M1 macrophage cytokines in the bronchoalveolar lavage fluid (BALF) of IPF patients, suggesting their active involvement in the disease’s pathogenic processes (265, 266). M1 macrophages also interact with alveolar epithelial cells, potentially inducing epithelial cell death and contributing to fibrosis through mechanisms that involve oxidative stress and cytokine-mediated damage (267). M1 macrophages also produce ROS and NO, which can cause tissue damage and further perpetuate the fibrotic process (268). Furthermore, M1 macrophages are implicated in the remodeling of the ECM by expressing and activating proteases like MMP-9, which degrade ECM components, contributing to the disrupted architecture characteristic of IPF lungs (269). The role of M1 macrophages in IPF is complex, as their pro-inflammatory signals are essential for fighting infections and clearing debris, yet these same signals can aggravate lung injury.

As IPF progresses, M2 macrophages become more prevalent, contributing to tissue fibrosis by inhibiting inflammatory responses and promoting tissue repair and fibrosis (270). M2 macrophages secrete a range of cytokines and growth factors, such as TGF-β and PDGF, which are directly involved in stimulating fibroblasts to produce collagen and other ECM proteins (20). Besides their direct role in fibrosis, M2 macrophages exhibit anti-inflammatory functions by producing cytokines such as IL-10 and TGF-β. But in IPF, the resolution of inflammation without adequate removal of fibrotic tissue can lead to excessive tissue remodeling and fibrosis (20). Moreover, M2 macrophages express specific enzymes and receptors like arginase-1 and CD206, which are involved in the orchestration of a tissue remodeling environment conducive to fibrosis (264). M2 macrophages interact with epithelial cells, endothelial cells, and other resident fibroblasts, facilitating a microenvironment conducive to fibrosis. Their ability to secrete MMPs and tissue inhibitors of metalloproteinases (TIMPs) further contributes to the imbalance between ECM production and degradation (271).

In IPF, there is an imbalance favoring M2 macrophages over M1 macrophages, which is more aligned with tissue repair and fibrosis rather than the resolution of inflammation. This imbalance may be due to the unique microenvironment in IPF that favors the conversion of macrophages towards an M2 phenotype, driven by factors such as IL-4, IL-13, and TGF-β found in the fibrotic lung.

Overall, in the early stages of IPF, M1 macrophages accumulate in large numbers in the lungs, releasing pro-inflammatory cytokines (such as TNF-α, IL-1β, and IL-6) and chemokines, which recruit other immune cells (such as neutrophils and lymphocytes) to the site of inflammation, leading to acute inflammation and damage to lung tissue. As IPF progresses, M2 macrophages gradually become dominant, especially at sites of fibrosis. M2 macrophages secrete pro-fibrotic factors such as TGF-β, which activate fibroblasts and promote the production and deposition of ECM components such as collagen, leading to fibrosis of lung tissue. In the late stages of IPF, the persistent activity of M2 macrophages results in extensive fibrosis, causing severe structural and functional disruption of lung tissue, ultimately leading to respiratory failure.

### 3.5 Targeting macrophage polarization as a new therapeutic approach

Recent studies have highlighted the importance of targeting macrophage polarization as a therapeutic strategy in IPF. Some existing anti-inflammatory and anti-fibrotic drugs can influence macrophage polarization states. Pirfenidone has been reported to inhibit M2 macrophage polarization via the TGF-β1/Smad3 pathway to ameliorate fibrosis (272). Modulating macrophage polarization has shown promise in alleviating IPF. For example, Plekhf1 was found to promote macrophage M2 polarization and increase pulmonary fibrosis through PI3K/AKT signaling. Hence, intratracheal administration of plekhf1 siRNA-loaded liposomes can effectively inhibit plekhf1 expression in the lungs and significantly protect mice from bleomycin (BLM)-induced lung injury and fibrosis while significantly reducing M2 macrophages in the lungs from accumulation (24). Studies have shown that Sart1 promotes M2 macrophage polarization and exacerbates pulmonary fibrosis by activating the STAT6/PPAR-γ signaling pathway. Liposomes loaded with Sart1 siRNA can effectively inhibit M2 macrophage polarization and reduce fibrosis (21). C/EBP homologous protein (Chop) deficiency mice exhibit protection against bleomycin-induced pulmonary fibrosis, which is attributed to a reduction in M2 macrophages and decreased secretion of TGF-β1 (273). Elamipretide reduces IPF-associated inflammation and fibrosis by inhibiting Nrf2-dependent NLRP3 inflammasome in macrophages (274). Fra-2-expressing macrophages promote pulmonary fibrosis in mice, exacerbating fibrosis by enhancing the polarization of M2 macrophages and the deposition of collagen. It is speculated that Fra-2 may be a potential therapeutic target for pulmonary fibrosis (275). Microcystin-LR inhibits the polarization of CD206^+^M2-like macrophages and alleviates pulmonary fibrosis by regulating GRP78-mediated endoplasmic reticulum stress response (178). Nicotinamide phosphoribosyltransferase (NAMPT) is the rate-limiting enzyme of the nicotinamide adenine dinucleotide (NAD) salvage biosynthesis pathway. Studies have shown that NAMPT exacerbates bleomycin-induced pulmonary fibrosis by promoting macrophage M2 polarization. Inhibition of NAMPT can reduce the generation of M2 macrophages and fibrosis (276). Furthermore, human pluripotent stem cell-derived macrophages and macrophage-derived exosomes have shown the potential to reduce lung fibrosis in animal models (46). The use of stem cell-derived macrophages and exosomes to treat IPF has potential clinical application prospects.

Macrophage polarization plays a key role in the occurrence and development of IPF. Understanding and regulating the balance of M1 and M2 macrophages is of great significance for exploring new treatments for IPF. Future studies should further explore the interaction between macrophages and other cell types (such as fibroblasts) to fully understand the pathological mechanisms of IPF. By analyzing and integrating multiple research literatures, it can be seen that macrophage polarization plays a dual role in IPF, and its regulation has an important impact on the progression and treatment of the disease. These findings provide a theoretical basis and practical guidance for the development of IPF treatment strategies targeting macrophage polarization.

## 4 Conclusions and outlook

The pathophysiology of IPF is marked by a complex interplay of cellular processes and inflammatory signals, with macrophage polarization playing a pivotal role. The dualistic nature of macrophage phenotypes (M1 and M2) underscores their significant yet contrasting contributions to the disease. M1 macrophages, known for their pro-inflammatory functions, are crucial in the early phases of IPF, potentially defending against pathogens and damaged cells, but their actions can exacerbate lung injury and inflammation. Conversely, M2 macrophages facilitate tissue repair and fibrosis, processes that, while essential for healing, can lead to excessive collagen deposition and lung stiffening characteristic of advanced IPF.

Research in recent years has provided valuable insights into how these macrophage phenotypes influence the fibrotic pathway, highlighting the complexity of their roles. M1 macrophages, through their secretion of inflammatory cytokines and enzymes, can damage lung tissue but also play a role in regulating fibrotic responses via antifibrotic mediators like NO. On the other hand, M2 macrophages promote ECM remodeling and fibroblast activation, contributing to the chronic progression of fibrosis. Their ability to modulate immune responses further adds to their significance in managing the inflammatory environment within fibrotic lung tissue.

Looking ahead, the therapeutic targeting of macrophage polarization presents a promising avenue for IPF treatment. Strategies that can modulate macrophage responses, tipping the balance away from M2-driven fibrosis without exacerbating M1-mediated tissue injury, could offer new hope for managing or even reversing IPF. However, the therapeutic targeting of macrophages in IPF must be approached with caution. Given the essential roles of macrophages in host defense and tissue homeostasis, indiscriminate suppression of macrophage function could lead to adverse effects, including increased susceptibility to infections and impaired wound healing. Thus, future therapies should aim for selective modulation of macrophage functions rather than broad suppression.

In conclusion, while the challenge of deciphering macrophage polarization in IPF is substantial, the potential rewards are profound. Increased knowledge of the functions of macrophages in IPF may result in innovative treatments that not only stop the advancement of the disease but also have the potential to reverse fibrotic alterations, leading to a substantial enhancement in patient results.

## Author contributions

ZG: Data curation, Formal analysis, Investigation, Writing – original draft, Writing – review & editing. YC: Investigation, Validation, Writing – review & editing. LM: Investigation, Writing – original draft. FH: Investigation, Writing – original draft. LX: Conceptualization, Validation, Visualization, Writing – original draft, Writing – review & editing.
